# A Rare Case of Pelvic Abscess Due to Spontaneous Non-traumatic Bladder Rupture

**DOI:** 10.7759/cureus.18913

**Published:** 2021-10-19

**Authors:** Kyosuke Inoguchi, Takashi Hongo, Hiromichi Naito, Atsunori Nakao

**Affiliations:** 1 Emergency Department, Okayama Saiseikai General Hospital, Okayama, JPN; 2 Emergency, Critical Care, and Disaster, Okayama University Graduate School of Medicine, Dentistry and Pharmaceutical Sciences, Okayama, JPN; 3 Emergency, Critical Care and Disaster, Okayama University Graduate School of Medicine Dentistry and Pharmaceutical Sciences, Okayama, JPN

**Keywords:** sepsis, pelvic abscess, conservative treatment, neurogenic bladder, bladder rupture

## Abstract

Spontaneous bladder rupture is an uncommon and life-threatening urological emergency, and early diagnosis is often challenging. Herein, we report a case of intraperitoneal bladder rupture in an 81-year-old male with neurogenic bladder-the case of intraperitoneal bladder rupture required late laparotomy for pelvic abscess following initial conservative treatment.

An eighty-one-year-old male presented to our emergency department with deterioration of consciousness, fever, and hematuria. He denied previous trauma history and had been treated for neurogenic bladder. Physical examination revealed signs of tenderness in the abdomen. A diagnosis of bladder rupture was made based on laboratory examination indicating renal failure and radiological imaging showing urinary ascites. Conservative management with a Foley catheter and antibiotics (meropenem administered 1 g/day) was initiated. On day seven after admission, the patient complained of abdominal pain and fever, and a diagnosis of pelvic abscess based on contrast-enhanced computed tomography and septic peritonitis was made. An emergency exploratory laparotomy for peritoneal drainage was performed. The postoperative course was uneventful, and the patient was discharged on day 29 after admission.

Urinary bladder rupture should always be considered as a differential diagnosis in patients presenting with free fluid in the abdomen, peritonitis, reduced urine output, and hematuria. Clinicians should be aware that secondary bacterial peritonitis can occur as a major complication of a ruptured urinary bladder.

## Introduction

Intraperitoneal bladder rupture occurs most commonly due to blunt/penetrating pelvic trauma, followed by iatrogenic causes, including surgeries, endoscopic procedures, and Foley catheter placement [1]. However, spontaneous intraperitoneal bladder rupture is quite rare due to difficulty in accurate and early diagnosis [2]. The definitive treatment for intraperitoneal bladder rupture typically involves surgical repair [1]. However, conservative treatment with urinary drainage is an alternative management strategy associated with favorable outcomes without any complications [3-7]. 

We herein report a case of spontaneous intraperitoneal bladder rupture in an 81-year-old Japanese man who was initially conservatively treated with Foley catheter drainage and antibiotics. 

## Case presentation

**Figure 1:**
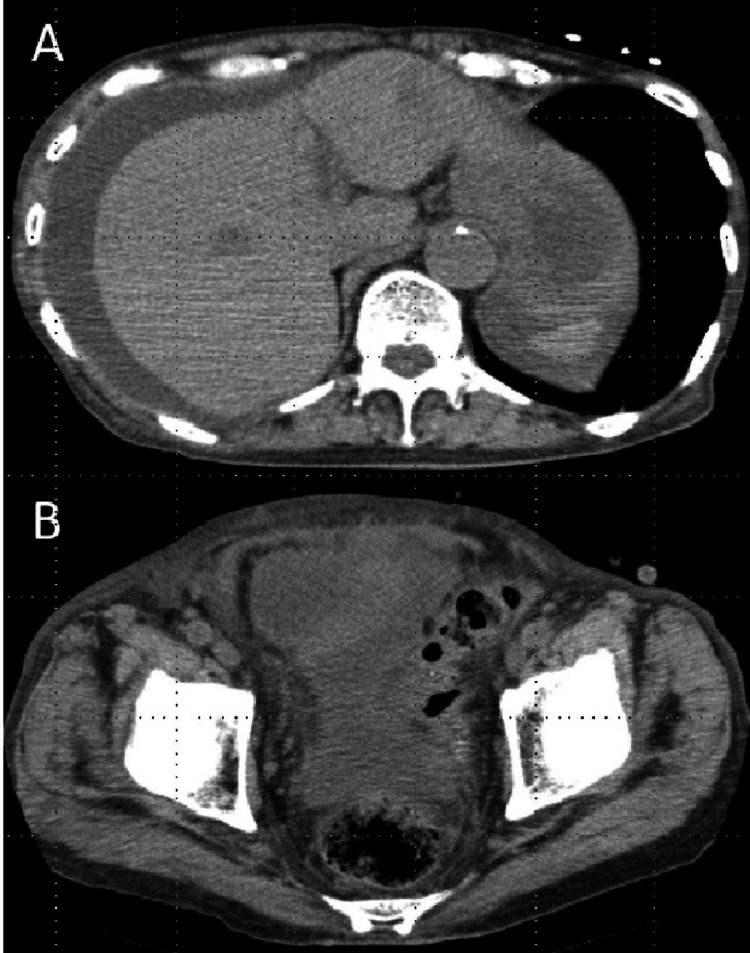
Abdominal computed tomography on admission Computed tomography revealed large amounts of hemorrhagic ascites in the abdominal cavity (A) and large amounts of ascites in the pelvic cavity (B).

**Figure 2:**
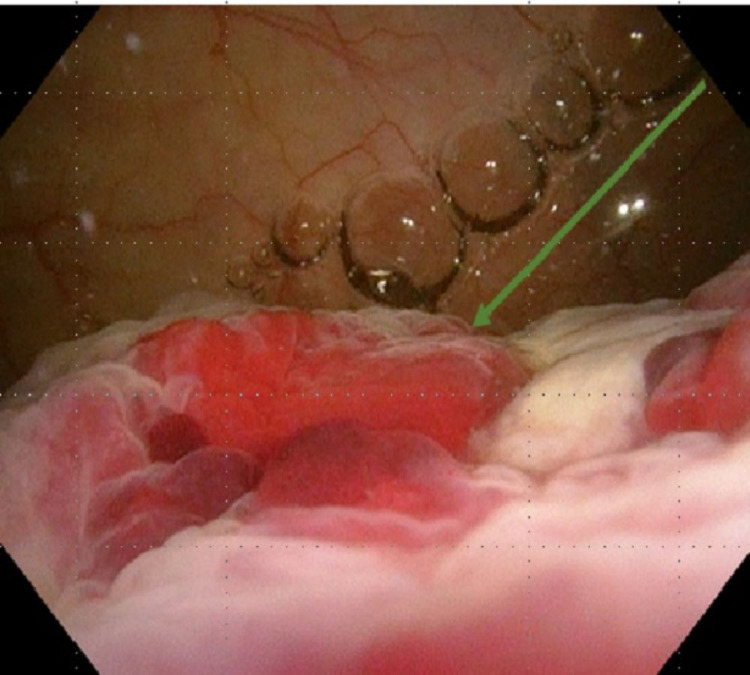
Cystoscopy on day three Cystoscopy revealed a fissure-like scar without visible large perforation defect from the dome to posterior wall of the bladder (arrow).

**Figure 3:**
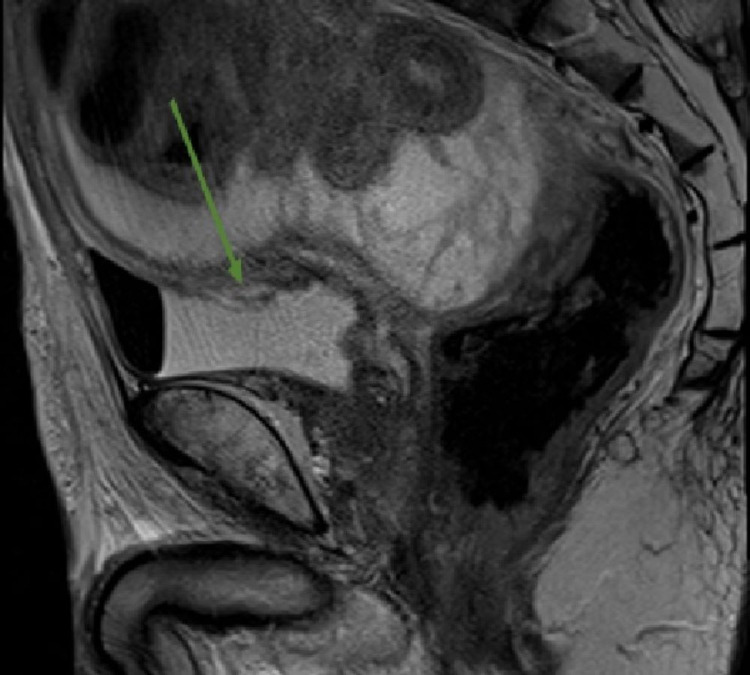
Pelvic magnetic resonance imaging on day four Pelvic T2-weighted magnetic resonance imaging revealed high signal intensity in the dome of the bladder, indicating rupture of the bladder wall (arrow).

**Figure 4:**
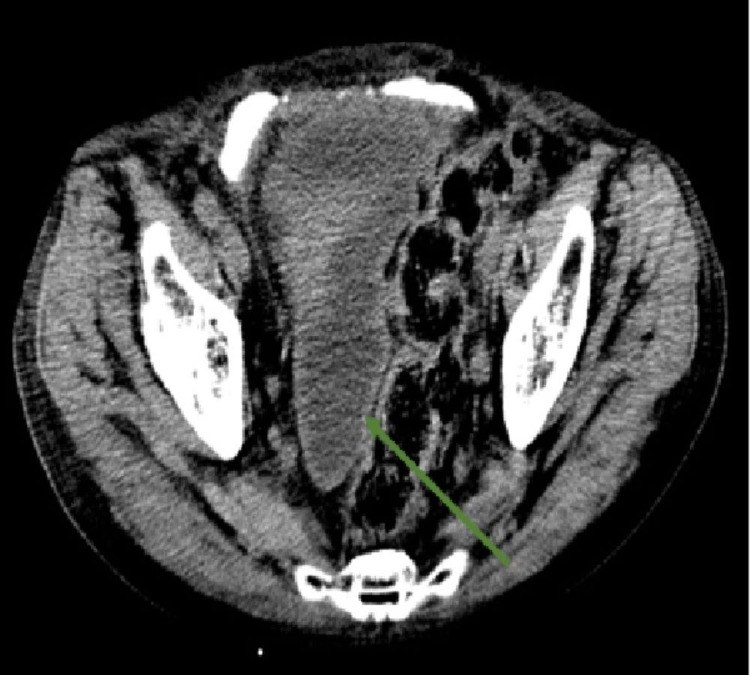
Computed tomography on day seven Contrast computed tomography revealed pelvic abscess around bladder (arrow).

An eighty-one-year-old man presented to our emergency department with disturbance of consciousness, fever, and hematuria for one day prior to the visit. His medical history included hypertension, cerebral hematoma, and neurogenic bladder, which were treated with oral fesoterodine (4 mg/day) and mirabegron (4 mg/day). He denied any previous abdominal/pelvic surgery or previous blunt abdominal trauma. His vital signs were a Glasgow Coma Scale score of nine (E2V2M5), blood pressure of 121/80 mmHg, heart rate of 120 bpm, the rectal body temperature of 38.8°C, respiratory rate of 30 breaths/min, and arterial oxygen saturation of 98% with oxygen delivery via a face mask (5 L/min). Physical examination revealed signs of tenderness and ascites in the bilateral lower quadrants. Laboratory results demonstrated leukopenia (white blood cell count of 28 × 10^2^ cells/dL), kidney injury as indicated by elevated blood urea nitrogen levels of 100 mg/dL, and creatinine levels of 8.65 mg/dL, and hyperglycemia (122 mg/dL). The procalcitonin and C-reactive protein levels were elevated to 76.40 ng/mL and 10.7 mg/dL. Urine analysis showed gross hematuria (red blood cells (RBCs) full number). Blood, urine, and ascites cultures obtained were positive for *Morganella morganii*, a facultative, anaerobic, gram-negative rod belonging to the Enterobacteriaceae family. Plain abdominal computed tomography (CT) revealed a hemorrhagic fluid collection in the peritoneal cavity without pneumoperitoneum (Figure 1). Hydronephrosis and ureter dilatation were absent. Based on these examinations, a diagnosis of urinary bladder rupture associated with sepsis was comprehensively made. Conservative management with a Foley catheter and antibiotics (meropenem administered 1 g/day) was initiated, as the patient was hemodynamically stable. He was polyuric and produced 2,900 mL of urine in the initial 24 h after admission. The serum levels of blood urea nitrogen decreased from 100 mg/dL to 73 mg/dL and creatinine from 8.65 mg/dL to 1.98 mg/dL on day two of admission. Cystoscopy to exclude neoplasm of the bladder demonstrated a fissure-like scar without visible large defect perforation from the dome to the posterior wall of the bladder (Figure 2). T2-weighted magnetic resonance imaging (MRI) revealed a focal defect area in the bladder muscular of the dome, indicating bladder rupture (Figure 3). At seven days after admission, the patient complained of severe abdominal pain with tenderness and fever. As the follow-up CT revealed pelvic abscess around the bladder (Figure 4), an exploratory laparotomy was performed. Intraoperative findings indicated severe bladder wall transmural inflammation and necrosis predominantly outside the bladder involving the perivesical adipose tissue without large defects. A leak test by indigo carmine injection in the bladder did not result in any leakage of blue-stained urine into the peritoneal cavity. Abdominal lavage and pelvic abscess drainage without bladder repair were performed. The patient’s general condition improved, and he was discharged to the rehabilitation center at a community hospital on the 29th day after admission without other complications.

## Discussion

Spontaneous bladder rupture without a traumatic episode is a rare urological emergency [8]. The predisposing factors causing bladder rupture without trauma include tumors, cystitis, connective tissue disorders, binge alcohol consumption, diverticulum rupture, bladder outlet obstruction, diabetes, radiation treatment, transvaginal delivery, and neurogenic bladder [5, 6, 9-11]. Bladder rupture is associated with increased intra-vesical pressure and weakening of the bladder wall and/or overdistension of the bladder with failure of neurosensory mechanisms [11]. In Japan, spontaneous bladder rupture is most commonly observed after pelvic radiotherapy [12]. However, the etiology in our patient was not related to a bladder tumor or radiotherapy. In our case, the patient was on anticholinergic medication fesoterodine, which decreases detrusor muscle contraction and has adverse effects on urinary retention [13]. These phenomena might be associated with bladder rupture with an increase in intravesical pressure or decreased strength of the bladder wall [8].

The clinical manifestations of bladder rupture include hematuria, anuria, abdominal pain, abdominal distention, voiding difficulty, and fever [10]. As the urine leaking into the abdominal cavity is resorbed into the systemic circulation, electrolyte and metabolic abnormalities described as “pseudo-renal failure” may become apparent [10, 14]. Our patient presented with impaired consciousness, hematuria, suprapubic pain, fever, and renal failure, which resembled peritonitis with sepsis. Thus, early and accurate preoperative diagnosis is quite challenging for emergency physicians.

Imaging modalities to identify bladder rupture include ultrasonography, CT scan, MRI cystography, and/or cystoscopy [3]. The ultrasonography may detect a bladder rupture, which may be collapsed bladder and contain little urine and massive ascites entire abdomen. Retrograde cystography can be used to assess suspected cases of bladder rupture, as it allows determination of the orientation of any contrast leakage from the urinary bladder into the peritoneal cavity and allows the determination and characterization of other pathologies [10]. However, there are reports of false-negative results in cases of small perforations and inadequate bladder distension with contrast material [15]. MRI is considered the ideal device for visualizing anatomical structures and intrinsic soft-tissue contrast. In our case, MRI revealed bladder wall defects and post-inflammatory changes following a rupture in the dome of the bladder. 

The American Urological Association guidelines recommend that uncomplicated extraperitoneal bladder injuries can be managed conservatively with catheter placement for several weeks [1, 3-7]. Extraperitoneal ruptures that do not heal after four weeks of catheter drainage, as well as intraperitoneal bladder ruptures, should be considered for surgical repair [1]. Our initial conservative treatment with urinary drainage and antibiotics was not successful, and a late laparotomy was required due to the development of secondary bacterial peritonitis and pelvic abscess. Clinicians should be aware that secondary bacterial peritonitis and sepsis are one of the major complications of a ruptured urinary bladder. 

During follow-up, patients with bladder rupture must be educated regarding bladder emptying to prevent overdistension and perforation without using anticholinergic agents. Sharing our experience may help emergency physicians diagnose and initiate treatment of bladder rupture early to avoid subsequent life-threatening complications.

## Conclusions

This entity of spontaneous bladder rupture should be kept in mind when an individual with neurogenic bladder presents with abdominal pain, ascites, and oliguric renal failure. As unrecognized and conservative unrepaired intraperitoneal bladder ruptures may lead to peritonitis, sepsis, and abscess, early diagnosis and treatment strategy is essential for a favorable outcome.
